# Non-Residential Preliminary Schools

**Published:** 1921-07-30

**Authors:** F. A. Sheldon

**Affiliations:** Guy's Trained Nurses' Institution, S.E. 1


*To the Editor of* The Hospital.

### Non-Residential Preliminary Schools.

*To the Editor of* The Hospital.

Sir,—In the article under this head in your issue of July 23, reference is made to a nursing sister as teacher. Is that necessary? At all women's colleges and at the large Polytechnics there are already the staff and the equipment for suitable lectures and demonstrations in anatomy, physiology, domestic and social science, and hygiene, etc., for the would-be probationer.

The General Nursing Council would provide its own syllabus, and machinery now in motion could be utilised, thus meeting an existing and immediate need.

Later in their career, and for more specialised instruction and hospital ethics, these nurse-students would come under the influence and teaching of that most finely conceived figure in modern nursing, the sister-tutor.—Yours, etc.,

F. A. Sheldon.

Guy's Trained Nurses' Institution, S.E. 1,
July 25, 1921.

